# Genetic mapping of QTLs for drought tolerance in chickpea (*Cicer arietinum* L.)

**DOI:** 10.3389/fgene.2022.953898

**Published:** 2022-08-19

**Authors:** Ashutosh Kushwah, Dharminder Bhatia, Rutwik Barmukh, Inderjit Singh, Gurpreet Singh, Shayla Bindra, Suruchi Vij, Bharadwaj Chellapilla, Aditya Pratap, Manish Roorkiwal, Shiv Kumar, Rajeev K. Varshney, Sarvjeet Singh

**Affiliations:** ^1^ Department of Plant Breeding and Genetics, Punjab Agricultural University, Ludhiana, Punjab, India; ^2^ Center of Excellence in Genomics and Systems Biology, International Crops Research Institute for the Semi-Arid Tropics (ICRISAT), Hyderabad, India; ^3^ Regional Research Station, Punjab Agricultural University, Faridkot, India; ^4^ Division of Genetics, ICAR-Indian Agricultural Research Institute, New Delhi, India; ^5^ Crop Improvement Division, ICAR- Indian Institute of Pulses Research, Kanpur, India; ^6^ International Center for Agricultural Research in the Dry Areas (ICARDA), Rabat Office, Rabat, Morocco; ^7^ Murdoch’s Centre for Crop and Food Innovation, State Agricultural Biotechnology Centre, Food Futures Institute, Murdoch University, Murdoch, WA, Australia

**Keywords:** genetic mapping, ddRAD-seq, single nucleotide polymorphism (SNP), quantitative trait locus (QTL), root system architecture

## Abstract

Chickpea yield is severely affected by drought stress, which is a complex quantitative trait regulated by multiple small-effect genes. Identifying genomic regions associated with drought tolerance component traits may increase our understanding of drought tolerance mechanisms and assist in the development of drought-tolerant varieties. Here, a total of 187 F_8_ recombinant inbred lines (RILs) developed from an interspecific cross between drought-tolerant genotype GPF 2 (*Cicer arietinum*) and drought-sensitive accession ILWC 292 (*C. reticulatum*) were evaluated to identify quantitative trait loci (QTLs) associated with drought tolerance component traits. A total of 21 traits, including 12 morpho-physiological traits and nine root-related traits, were studied under rainfed and irrigated conditions. Composite interval mapping identified 31 QTLs at Ludhiana and 23 QTLs at Faridkot locations for morphological and physiological traits, and seven QTLs were identified for root-related traits. QTL analysis identified eight consensus QTLs for six traits and five QTL clusters containing QTLs for multiple traits on linkage groups CaLG04 and CaLG06. The identified major QTLs and genomic regions associated with drought tolerance component traits can be introgressed into elite cultivars using genomics-assisted breeding to enhance drought tolerance in chickpea.

## Introduction

Chickpea (*C. arietinum* L.) is an important cool-season legume crop cultivated largely in the semi-arid and arid regions of Asia and sub-Saharan Africa (Gaur et al., 2012). It is a diploid (2n = 2x = 16) and self-pollinated crop with a genome size of ∼738 Mb (Varshney et al., 2013a) and serves as a rich source of nutrients such as proteins (23%), carbohydrates (40%), vitamins, essential amino acids, and free from anti-nutritional factors (Jukanti et al., 2012; Roorkiwal et al., 2021). In spite of its economic importance and its role in improving human health, chickpea production is falling short of meeting the dietary needs of the burgeoning human population, mainly because of low productivity due to biotic and abiotic constraints (Thudi et al., 2014; Roorkiwal et al., 2020; Kushwah et al., 2021a). Among the abiotic stresses, drought alone causes up to 60% of annual yield losses in chickpea (Sabaghpour et al., 2006; Toker et al., 2007; Varshney et al., 2010; Hajjarpoor et al., 2018; Barmukh et al., 2022). The impact of global warming and climate change has emphasized researchers’ need to study the effect of drought stress on crop development and yield. Thus, it has become imperative to develop cultivars, which can attain their maximum potential under drought stress or rainfed environments.

Usually, drought stress adversely affects the plants through a transient or terminal drought (Li et al., 2018; Varshney et al., 2021). In general, it is a terminal drought that can terminate or reduce the reproductive phase to drastically reduce the crop yield. Drought tolerance is a complex quantitative trait affected by significant genotype by environment (G × E) interactions (Kashiwagi et al., 2008; Varshney et al., 2014; Barmukh et al., 2022), which hampers direct selection of genotypes with higher yield under drought conditions. Drought stress is well-known for reducing crop growth, thus affecting yield components, such as total biomass, pod number, seed number, seed weight, seed quality, and yield per plant (Toker et al., 2007; Krishnamurthy et al., 2013). Understanding the genetic basis of drought tolerance is difficult due to multiple underlying mechanisms used by plants to survive, such as drought escape, drought avoidance, and drought tolerance (Tuberosa & Salvi, 2006). The close association between several morphological and physiological traits (e.g., crop growth rate, leaf area index, canopy temperature depression, shoot biomass, phenology, etc.) with grain yield under drought was revealed in a previous study (Purushothaman et al., 2016). Early flowering in chickpea can be advantageous for enhancing the seed yield by shortening the vegetative phase and completing the crop life cycle prior to the onset of terminal drought stress (Kushwah et al., 2020b). Several other physiological traits, such as membrane permeability index, photosynthetic efficiency, relative leaf water content, chlorophyll content, proline accumulation, and ABA content, and morphological traits, such as days to germination, days to flowering, plant height, biomass, and 100-seed weight, have been proposed for the selection of drought-tolerant chickpea genotypes (Gaur et al., 2008; Kashiwagi et al., 2013; Purushothaman et al., 2016; Maqbool et al., 2017).

To further understand the drought tolerance mechanisms, screening of chickpea germplasm has led to the selection of genotypes with extensive root systems and better productivity under drought stress (Kashiwagi et al., 2005). Phenotypic attributes of the root system have gained more importance as they are expected to have a direct effect on transpiration in plants under drought stress (Ye et al., 2018). A profuse root system is expected to extract more soil water than a less extensive root system under drought stress (Zaman-Allah et al., 2011). Detailed studies on various root traits are difficult due to low heritability and complex mechanisms of these traits, variable expression across diverse soil environments, and the labor-intensive nature of the studies (Gaur et al., 2008; Varshney et al., 2011). Association studies have revealed positive associations (Kell, 2011; Bishopp & Lynch, 2015) and negative or neutral associations (Zaman-Allah et al., 2011; Schoppach et al., 2013) of profuse root systems with grain yield.

Considering the challenges associated with breeding drought-tolerant varieties, the identification of quantitative trait loci (QTLs) for drought tolerance component traits can be a judicious approach in a chickpea breeding program. For instance, QTLs identified in previous studies have helped in dissecting the genetic basis of drought tolerance-related traits in chickpea (Varshney et al., 2014; Sivasakthi et al., 2018). Furthermore, during the last decade, chickpea researchers have deciphered the chickpea genome (Varshney et al., 2013a; Jain et al., 2013) and developed several genomic (Roorkiwal et al., 2017; Roorkiwal et al., 2020) and transcriptomic resources (Hiremath et al., 2011; Kudapa et al., 2014; Mashaki et al., 2018) that have transformed chickpea from an “orphan crop” to a “genomics resource-rich crop” for faster genetic gains in chickpea (Varshney, 2016). Rapid advances in next-generation sequencing technologies have enabled the use of sequencing-based genotyping platforms for unraveling the genetic basis of several complex traits. The double digestion restriction site-associated DNA sequencing (ddRAD-seq) approach, developed by Peterson et al. (2012), can adjust the number of fragments by utilizing two different restriction enzymes (Puritz et al., 2014) and exclusively uses size selection for recovering the appropriate number of regions, which are arbitrarily distributed throughout the genome and maximizes the ability of multiplexing of numerous samples.

Due to the comparatively narrow genetic base of chickpea (Stephens et al., 2014) and relatively low levels of polymorphism, interspecific RIL populations from *C. arietinum* and *C. reticulatum* have been used efficiently for genetic studies (Singh et al., 2008; Barmukh et al., 2021a). The amount of polymorphism varied from 16% to 36% in an interspecific mapping population compared to only 9.5% in an intraspecific mapping population (Nayak et al., 2010). Interspecific populations have been proven to be useful for the construction of high-density genetic maps as well as trait dissection (Thudi et al., 2011; Roorkiwal et al., 2017; Barmukh et al., 2021a).

In the present study, an interspecific mapping population derived from a cross between GPF 2 (*C. arietinum*) and ILWC 292 (*C. reticulatum*) was used to identify key genomic regions associated with drought tolerance component traits. Some promising RILs were also detected for yield, morpho-physiological, and root-related traits under rainfed conditions for use in breeding programs. Identification and development of markers for drought tolerance component traits will be useful for deploying genomics-assisted breeding for the development of drought-tolerant improved chickpea varieties.

## Materials and methods

### Plant material and experimental sites

A set of 187 RILs (F_8_ generation) segregating for drought tolerance component traits was developed from an interspecific cross of drought-tolerant genotype GPF 2 (*C. arietinum*) with drought-sensitive accession ILWC 292 (*C. reticulatum*) using a single seed descent method as described in Kushwah et al. (2021b). Chickpea cultivar GPF2 is a semi-erect, medium-tall cultivar recommended for cultivation in Punjab state and in the North-Western Plains Zone of India. Apart from its drought tolerance characteristics, GPF2 is also resistant to *Fusarium* wilt and *Ascochyta* blight (Kushwah et al., 2021c). The accession ILWC 292 (*C. reticulatum*) has a semi-prostrate growth habit. It is sensitive to drought and susceptible to *Ascochyta* blight but resistant to botrytis grey mold and chickpea cyst nematode. Although ILWC 292 is a drought-sensitive genotype, it possesses some desirable drought tolerance-related traits such as higher root length density, root to shoot ratio, and membrane permeability index (Singh et al., 1996).

The RIL population and the parental genotypes were grown during the 2017–18 crop season, in an alpha lattice design (17 × 12) under irrigated (non-stress) and rainfed (drought-stress) conditions at two locations in India (Ludhiana and Faridkot), with three replications. Each RIL was planted in 2 m long paired rows with 30 cm × 10 cm spacing. The Ludhiana (30.9010°N, 75.8573°E) and Faridkot (30.6769°N, 74.7583°E) sites are categorized as semi-arid sub-tropical and semi-arid dry regions, respectively. Both sites consist of loamy sand with 59.8% sand and 16.5% clay (Typic Ustorthents). The average annual rainfall is 700 mm at Ludhiana and 450 mm at Faridkot, of which more than 70% occurs from July to September (Yadav et al., 2007; Kang et al., 2009).

### Phenotyping for morphological and physiological traits under field conditions

Sowing under field conditions was performed during November–April of the 2017–18 crop season on the residual soil moisture, which was sufficient for good germination, as recommended for chickpea sowing in this region. Soil moisture was measured at the time of sowing, after 70, 90, 110, and 130 days of sowing, and at the time of maturity in irrigated and rainfed conditions at both the locations. In the case of rainfed plots, the soil moisture was ideal for drought conditions for chickpea crop. As a result of the drastic reduction in soil moisture content at 90, 110, and 130 days after sowing in rainfed plots as compared to irrigated plots, sufficient drought stress was induced at the reproductive (flowering and pod formation) and pod development stages at both the locations.

Phenotypic data were collected for 21 traits including 12 morphological and physiological traits, namely, days to germination (DG), days to flowering initiation (DFI), days to 50% flowering (DFF), days to 100% flowering (DHF), plant height (PH), number of pods per plant (NPP), biomass (BIO), yield (YLD), 100-seed weight (HSW), harvest index (HI), membrane permeability index (MPI), relative leaf water content (RLWC), and nine root-related traits. Five randomly selected plants were used to record observations on PH, NPP, BIO, and YLD in each plot. The data for DG, DFI, DFF, DHF, and HSW were recorded on a plot basis. HI was calculated as follows:

HI =(seed yield/total shoot biomass)×100. (1)

MPI was determined according to the method of Premchandra et al. (1990), and modified by Sairam (1994), as follows:

MPI=[1- (C1/C2)]×100, (2)

where C1 is the initial electrical conductivity at 40°C and C2 is the final electrical conductivity at 100°C.

RLWC was calculated using the formula (Slavik, 1974):

RLWC(%)=[(FW-DW)/(TW-DW)]×100, (3)

where FW is the fresh weight, DW is the dry weight, and TW is the turgid weight.

### Phenotyping for root-related traits

A total of nine traits, i.e., root length (RL), shoot length (SL), root to shoot ratio (RSR), root length density (RLD), fresh root weight (FRW), fresh shoot weight (FSW), root dry weight (RDW), shoot dry weight (SDW), and the ratio of root dry weight to total plant dry weight (RDW/TDW) were recorded in the present study. A polyvinyl chloride (PVC) pipe-based cylinder culture approach was used for phenotyping root-related traits. Chickpea plants were grown in PVC cylinders (18 cm in diameter and 120 cm in height) with three replications. The PVC cylinders, except for the top 15 cm, were filled with an equi-mixture (w/w) of vertisol and sand, mixed with 0.07 g·kg^−1^ diammonium phosphate. The soil water content of the mixture was equilibrated to 70% field capacity to create conditions similar to those in the field at sowing when the soil is not fully saturated with water. A mixture of soil and sand was used to decrease the soil bulk density and facilitate root growth and extraction. Sampling was carried out at 35 days after sowing (DAS), avoiding physically damaged plants. This is because maximum variations in root-related traits among genotypes were best detected at 35 DAS and variation decreased after 41 DAS (Krishnamurthy et al., 1996).

Root samples were collected using steel soil-coring tubes (50 mm in diameter) to a depth of 120 cm at the flowering stage. Each RIL sample comprised three soil cores, which were pooled to increase the sample size. The soil cores for each sample were soaked overnight in water, and the roots were recovered by passing the suspension through a 2 mm wire mesh sieve. Chickpea roots were separated manually from the debris and weed roots. Total RL and FRW were measured, and roots were then oven-dried at 70°C for 72 h before measuring RDW. Likewise, FSW was measured, and then shoots were oven-dried at 70°C before measuring SDW. The RLD (cm^−3^) was measured as root length (cm)/volume of soil core (cm^3^), while the root to shoot ratio (RSR) was calculated using root and shoot lengths.

### Statistical analysis

Analysis of variance (ANOVA) was computed for individual environments using mixed model analysis to estimate the contribution made by each factor to the total variation using SAS software version 9.3 (SAS Institute, Cary, NC). The data from irrigated and rainfed conditions were used to estimate the best linear unbiased predictors (BLUPs) using the residual maximum likelihood algorithm (ReML) in the R package lmer (Bates et al., 2015). BLUPs were estimated for all 21 morpho-physiological traits, and scatter plots were drawn for all the traits using BLUPs to find the correlation between the two locations evaluated, i.e., Ludhiana and Faridkot.

### QTL analysis

The RIL population was genotyped with ddRAD-seq (Peterson et al., 2012), which uses *PstI* and *MspI* restriction enzymes (Thermo Fisher Scientific, MA, United States). The ddRAD-seq data analysis of RILs for SNP discovery and development of linkage map was performed as described previously (Kushwah et al., 2021c).

QTL analysis was performed with the composite interval mapping (CIM) method executed in the Windows QTL Cartographer V2.5 software package (Wang et al., 2007). The CIM analysis was conducted using forward and backward stepwise regression. For each trait, experiment-wise significance thresholds (*p* ≤ 0.05) were determined with 1,000 permutations for QTL detection. The position of the QTLs was identified on the basis of its logarithm of odds (LOD) peak location with a 95% confidence interval. The percentage of phenotypic variance and additive effect described by QTLs was also estimated. QTLs explaining total phenotypic variance (PVE) >10% were classified as major-effect QTLs, whereas those with PVE <10% were regarded as minor-effect QTLs (Varshney et al., 2014). The phenotypic contribution (R^2^) was estimated as the percentage of variance explained by each QTL in proportion to the total phenotypic variance, while the additive effect was estimated to find the positive or negative effect for the respective trait.

## Results

### Phenotypic variation among the RILs and parental genotypes

The RILs along with parent genotypes were evaluated under irrigated (non-stress) and rainfed (drought-stress) conditions at Ludhiana and Faridkot locations in India. Significant variation was observed among the RILs and their parents for multiple morphological, physiological, and root-related traits under irrigated and rainfed conditions (Tables 1, 2). Phenotyping data analysis for all the traits in parental lines depicted highly significant differences under irrigated and rainfed conditions. All of the measured morphological, physiological, and root-related traits were significantly affected by drought stress, except for HI, RL, RLD, and FRW. Under rainfed conditions, the RILs had slightly higher mean values (8.68%) for DG, while values for DFI, DFF, and DHF decreased by 16.11, 14.48, and 13.76%, respectively, when compared to irrigated conditions. Similarly, RILs possessed significantly lower mean values for PH (30.91%), NPP (42.50%), BIO (40.15%), and YLD (44.18%) under rainfed conditions. A moderate reduction in the mean performance of HSW (18.28%) and RLWC (19.53%) was observed under rainfed conditions relative to irrigated conditions. The rainfed conditions significantly increased the mean values of RILs for MPI (16.34%), while HI was least affected by water deficit with only a 0.09% reduction, relative to the irrigated conditions (Table 1). In the case of root-related traits, water deficit significantly reduced the mean values of RILs for SL (13.93%), FSW (25.40%), and SDW (14.05%), and slightly reduced for FRW (0.82%), relative to the irrigated conditions. A significant increase in the mean performance of RILs for RSR (18.40%), a moderate increase for RDW (6.09%) and RRDWTDW (9.30%), and a slight increase for RL (1.39%) and RLD (0.21%) were observed under rainfed conditions relative to the irrigated conditions (Table 2).

**TABLE 1:** Mean performance of chickpea RIL population for various morphological and physiological traits under irrigated (IR) and rainfed (RF) conditions (Ludhiana and Faridkot pooled).

Trait	Env	ILWC 292 (susceptible parent)	GPF 2 (tolerant parent)	Contrast analysis between parents	Mean (RILs)	CV	Range	Genotypic variance	G × L variance	H^2^ (broad sense)
DG	IR	12.34	8.12	44.33**	9.33	5.05	8.12–12.34	1.50**	0.51	56.40
	RF	13.70	8.53	194.89**	10.14	10.10	8.27–13.89	2.35**	0.15	42.40
DFI	IR	90.33	82.80	78.87**	85.99	2.01	78.91–90.33	1.96**	0.15	35.20
	RF	78.15	70.10	28.44**	72.14	9.45	55.53–87.61	15.24**	0.61	88.40
DFF	IR	94.26	86.46	171.50**	89.46	2.08	82.35–94.26	2.24**	0.14	25.10
	RF	82.14	73.57	28.91**	76.51	8.75	60.19–91.55	25.76**	0.52	88.60
DHF	IR	98.08	89.78	171.50**	93.10	1.96	86.67–98.08	2.28**	0.20	29.70
	RF	84.93	76.45	30.18**	80.33	8.20	64.44–95.80	16.30**	0.59	88.40
PH	IR	42.42	58.87	65.43**	45.68	10.47	33.82–58.87	4.05**	3.85**	79.40
	RF	23.55	48.52	538.06**	31.56	26.12	12.74–48.52	21.30**	4.58**	89.00
NPP	IR	43.53	68.54	133.10**	47.39	21.75	25.13–75.07	18.64**	5.82**	85.30
	RF	18.09	43.71	169.97**	27.25	35.25	12.69–50.09	37.02**	4.12**	89.50
BIO	IR	76.78	113.32	55.17**	81.33	15.47	51.55–113.70	10.35**	4.12**	84.20
	RF	40.72	77.51	450.56**	48.68	33.28	18.68–83.48	35.06**	6.58**	89.70
YLD	IR	27.91	49.74	232.07**	32.14	27.07	14.13–54.69	18.16**	6.12**	88.00
	RF	11.78	36.77	200.39**	17.94	50.90	7.31–45.79	30.72**	9.12**	89.20
HSW	IR	11.27	16.18	1,629.73**	14.22	16.11	9.79–18.42	20.41**	18.35**	90.40
	RF	10.33	16.09	714.84**	11.62	20.33	7.16–17.10	25.40**	9.89**	90.10
HI	IR	36.63	43.55	15.81**	38.98	16.48	22.49–52.86	12.52**	5.31**	86.40
	RF	28.89	47.96	54.76**	35.37	19.94	23.55–56.56	8.36**	7.53**	87.50
MPI	IR	42.23	28.81	150.71**	39.42	12.12	28.70–50.76	15.49**	8.51**	88.70
	RF	50.24	36.17	91.06**	47.12	13.02	31.56–58.10	19.61**	3.08**	88.70
RLWC	IR	65.28	88.31	178.91**	74.85	9.37	59.06–89.94	6.07**	10.17**	86.60
	RF	48.11	78.16	429.24**	60.23	15.97	44.80–79.65	14.39**	8.96**	88.90

**Highly significant at 1% probability level, DG, days to germination; DFI, days to flowering initiation; DFF, days to 50% flowering; DHF, days to 100% flowering; PH, plant height (cm); NPP, number of pods per plant; BIO, biomass per plant (gm); YLD, yield per plant (gm); HSW, 100-seed weight (gm); HI, harvest index (%); MPI, membrane permeability index; RLWC, relative leaf water content (%); Env, environment; CV, coefficient of variation; G × L, genotype by location interaction; H^2^, broad-sense heritability.

**TABLE 2:** Mean performance of chickpea RIL population for root-related traits under irrigated (IR) and rainfed (RF) conditions.

Trait	Env	ILWC 292 (susceptible parent)	GPF 2 (tolerant parent)	Mean (RILs)	CV	Range	Genotypic variance	G × E variance	H^2^ (broad sense)
RL	IR	84.82	109.60	90.84	17.08	57.37–128.17	24.25**	1.69**	96.30
	RF	88.98	125.71	92.10	17.86	56.10–127.46	20.23**		95.40
SL	IR	26.60	35.65	28.72	19.22	16.84–47.99	7.20**	2.13**	88.00
	RF	17.07	31.82	24.72	21.95	13.56–41.36	10.81**		92.20
RSR	IR	3.19	3.08	3.26	18.92	1.90–4.89	4.50**	1.74**	78.80
	RF	5.20	3.97	3.86	20.50	1.99–6.94	6.40**		85.50
RLD	IR	9.14	6.71	9.33	23.97	5.67–13.85	12.29**	7.26**	92.60
	RF	10.52	6.64	9.35	20.21	4.67–14.24	28.89**		96.90
FRW	IR	8.99	11.46	9.72	17.06	6.37–13.73	10.46**	1.61**	91.30
	RF	7.93	12.26	9.64	17.39	5.71–13.62	17.26**		94.90
FSW	IR	9.40	15.04	11.93	27.94	7.68–25.04	33.77**	5.35**	97.40
	RF	5.71	17.32	8.90	43.68	4.79–24.92	31.96**		97.10
RDW	IR	2.00	3.00	2.30	28.62	1.17–3.85	10.28**	3.83**	91.20
	RF	1.39	4.69	2.44	45.20	0.37–5.80	15.49**		94.20
SDW	IR	2.49	3.61	2.99	22.43	2.13–5.47	31.62**	5.03**	97.20
	RF	1.97	4.25	2.57	30.18	1.60–5.60	26.99**		96.70
RDW/TDW	IR	0.45	0.46	0.43	12.58	0.25–0.55	5.09**	3.22**	81.30
	RF	0.42	0.52	0.47	21.63	0.18–0.74	8.46**		89.20

**Highly significant at 1% probability level, RL, root length; SL, shoot length; RSR, root-shoot ratio; RLD, root length density; FRW, fresh root weight; FSW, fresh shoot weight; RDW, root dry weight; SDW, shoot dry weight; RDW/TDW, ratio of root dry weight to total plant dry weight; Env, environment; CV, coefficient of variation; G × E, genotype by environment interaction; H^2^, broad-sense heritability.

The pooled ANOVA for all the morphological and physiological traits showed highly significant differences between genotypes at both locations in the irrigated and rainfed conditions. Significant differences were also observed for genotype × environment (G × E) interactions for these traits, except for DG, DFI, DFF, and DHF (Table 1). The scatter plots showed a highly significant relationship between locations for most of the traits except PH, HI, and RLWC which showed a moderately high correlation coefficient (Supplementary Figure S1). In the case of root-related traits, pooled ANOVA for variation in genotypes and G × E interactions showed highly significant differences for the RIL population under irrigated and rainfed conditions at both locations (Table 2). Under irrigated conditions, PH, NPP, BIO, YLD, HSW, HI, MPI, and RLWC had higher broad-sense heritability (79.40–90.40%), while low to moderate heritability (25.10–56.40%) was observed for DG, DFI, DFF, and DHF. By contrast, under rainfed conditions, all traits had high heritability (87.50–90.10%), except for DG (42.40%) (Table 1). The root-related traits had high broad-sense heritability under both irrigated (78.80–97.40%) and rainfed conditions (85.50–97.10%) (Table 2). A significantly high correlation coefficient between the irrigated and rainfed conditions was observed for all root-related traits, except RLD (Supplementary Figure S2).

### Selection of promising lines

A total of 17 out of 187 RILs were found to be highly promising for yield, morpho-physiological, and root-related traits under rainfed conditions (Tables 3, 4). Of these 17 lines, five lines showing early flowering were also promising for yield-related traits (Table 3).

**TABLE 3:** List of promising recombinant inbred lines for various morphological and physiological traits under rainfed conditions (Ludhiana and Faridkot pooled).

RIL no.	DG	DFI	DFF	DHF	PH	NPP	BIO	YLD	HSW	HI	MPI	RLWC
75	8.38	**57.48**	**62.11**	**66.83**	44.43	50.09	81.46	45.79	15.70	56.56	31.88	78.10
81	8.27	**57.38**	**61.84**	**66.43**	45.06	47.98	76.99	42.68	17.06	56.02	32.58	77.59
154	9.30	69.79	73.67	77.34	46.36	49.71	83.48	42.05	16.50	50.87	31.56	79.37
41	9.61	73.00	77.19	81.06	40.94	46.28	73.21	38.92	16.22	53.97	34.47	75.95
26	10.69	69.17	73.75	77.02	47.34	50.07	79.78	38.67	17.10	48.96	35.21	76.53
56	9.94	**59.07**	**63.86**	**68.20**	41.92	43.45	75.33	37.12	15.24	49.38	35.52	78.02
16	10.66	83.69	87.96	91.67	39.00	43.97	74.12	37.05	14.66	50.35	34.74	76.78
13	9.96	63.06	68.37	72.38	41.99	45.84	77.04	36.90	15.55	48.04	37.35	74.81
9	9.02	67.12	71.59	75.79	42.78	40.51	69.77	36.28	15.47	52.37	36.70	75.85
77	9.06	**57.32**	**61.63**	**66.56**	45.14	44.35	79.20	36.16	16.96	45.80	37.01	77.59
7	9.34	**57.79**	**62.58**	**65.77**	46.52	43.00	73.74	35.83	15.66	48.75	36.88	72.57
80	8.68	61.83	66.72	70.62	46.26	43.73	73.65	35.68	13.89	49.47	36.92	75.94
15	9.39	76.98	80.78	84.57	39.41	40.31	70.91	35.60	16.84	50.47	37.82	76.97
180	10.45	79.47	83.64	87.30	44.12	45.95	73.39	35.14	14.04	48.17	36.63	71.25
70	9.15	69.19	73.85	77.90	45.53	46.17	81.41	34.81	15.72	43.02	38.48	76.67
62	9.57	67.85	71.93	76.16	44.93	42.37	76.18	34.68	14.73	45.60	37.94	77.88
24	9.84	66.00	70.41	73.80	43.45	42.47	71.71	34.43	15.24	48.04	38.28	79.65
GPF2	8.53	70.10	73.57	76.45	48.52	43.71	77.51	36.77	16.09	47.96	36.17	78.16
ILWC292	13.70	78.15	82.14	84.93	23.55	18.09	40.72	11.78	10.33	28.89	50.24	48.11

Note: Promising recombinant inbred lines showing early flowering are marked as bold.

**TABLE 4:** List of promising recombinant inbred lines for various root-related traits under rainfed conditions at Ludhiana.

RIL no.	RL	SL	RSR	RLD	FRW	FSW	RDW	SDW	RDW/SDW
75	127.46	41.36	3.05	5.72	13.62	8.45	5.18	2.47	0.69
81	84.89	27.81	3.12	10.53	9.31	14.94	2.18	3.80	0.38
154	106.69	27.37	3.89	8.83	11.11	14.70	3.21	3.66	0.46
41	105.03	23.15	4.63	14.12	11.12	15.36	3.19	3.88	0.45
26	122.08	34.71	3.50	5.83	13.10	12.99	4.70	3.41	0.58
56	87.42	21.53	4.08	12.55	9.41	8.10	2.36	2.42	0.50
16	114.57	33.74	3.37	4.67	12.24	19.96	4.27	4.65	0.47
13	114.54	29.34	3.90	8.93	12.41	11.07	4.47	3.02	0.59
9	90.08	23.55	3.90	12.97	9.51	7.04	2.45	2.21	0.52
77	93.47	34.22	2.73	10.63	10.29	13.70	2.62	3.58	0.43
7	97.32	23.26	4.18	11.70	10.05	8.58	2.72	2.54	0.51
80	100.89	26.26	3.88	9.20	10.65	11.83	3.10	3.17	0.50
15	109.93	28.63	3.91	8.95	11.70	9.27	3.81	2.67	0.57
180	84.67	28.05	3.05	8.78	9.21	6.84	2.21	2.18	0.50
70	124.62	39.99	3.05	5.62	13.41	23.68	5.08	5.22	0.49
62	121.75	26.56	4.59	6.03	12.72	12.37	4.78	3.30	0.60
24	116.73	34.18	3.36	9.25	13.00	12.84	4.65	3.39	0.57
GPF2	125.71	31.82	3.97	6.64	12.26	17.32	4.69	4.25	0.52
ILWC292	88.98	17.07	5.20	10.52	7.93	5.71	1.39	1.97	0.42

### QTLs for drought tolerance component traits

**FIGURE 1:**
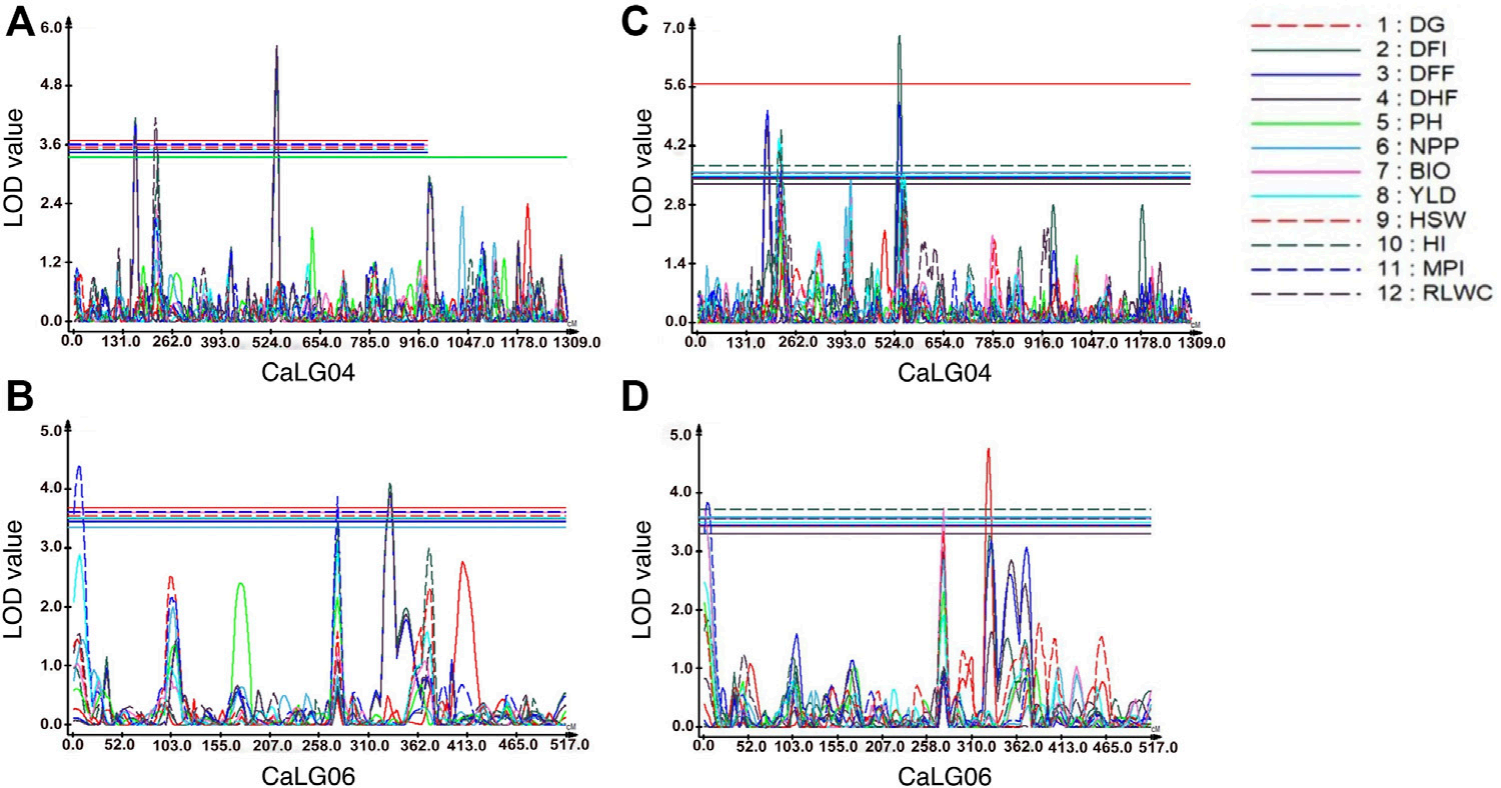
Genomic regions with QTLs for morphological and physiological traits. For traits evaluated at Faridkot, **(A)** QTLs for days to flowering initiation (DFI), days to 50% flowering (DFF), days to 100% flowering (DHF), harvest index (HI), and relative leaf water content (RLWC) were mapped on CaLG04; while **(B)** QTLs for days to flowering initiation (DFI), days to 50% flowering (DFF), days to 100% flowering (DHF), yield (YLD), harvest index (HI), and membrane permeability index (MPI) were mapped on CaLG06. For traits evaluated at Ludhiana, **(C)** QTLs for days to flowering initiation (DFI), days to 50% flowering (DFF), days to 100% flowering (DHF), yield (YLD), harvest index (HI), membrane permeability index (MPI), and relative leaf water content (RLWC) were mapped on CaLG04; whereas **(D)** QTLs for days to germination (DG), days to flowering initiation (DFI), days to 50% flowering (DFF), number of pods per plant (NPP), biomass per plant (BIO), 100-seed weight (HSW), membrane permeability index (MPI), and relative leaf water content (RLWC) were mapped on CaLG06.

A total of 1,365 polymorphic SNPs were used for linkage map construction (Kushwah et al., 2021c). Genotypic data on 1,365 informative SNPs, linkage map distances, and BLUP values for 21 traits evaluated at two locations were used to identify QTLs for drought tolerance component traits. A total of 31 QTLs at Ludhiana and 23 QTLs at Faridkot were identified for 12 morphological and physiological traits, except root-related traits, evaluated under irrigated and rainfed conditions (Tables 5, 6; Figure 1; Supplementary Figure S3). Out of 31 QTLs identified at Ludhiana, 14 were classified as major-effect QTLs and 17 as minor-effect QTLs. Likewise, out of 23 QTLs identified at Faridkot, 15 were major-effect QTLs and seven were minor-effect QTLs.

**TABLE 5:** Summary of QTLs associated various morphological and physiological traits evaluated at Ludhiana.

S. no.	Trait	Chr	QTL name	LOD	Additive effect	R^2^ (%)	TR^2^	Left flanking marker position (cM)	Right flanking marker position (cM)	Left flanking marker	Right flanking marker
1	DG	6	*qdg-01*	4.77	0.1178	10.28	0.2061	321.15	328.78	CNC_021165.1.18056125	CNC_021165.1.513801
		7	*qdg-02*	4.53	0.1425	18.37	0.3323	541.62	552.69	CNC_021166.1.34922231	CNC_021166.1.15786786
2	DFI	**4**	***qdfi-01***	**6.85**	**0.8494**	**17.47**	**0.3047**	**532.51**	**545.63**	**CNC_021163.1.11351447**	**CNC_021163.1.12812015**
		**6**	***qdfi-02***	**3.29**	**0.5900**	**6.41**	**0.2614**	**321.15**	**328.78**	**CNC_021165.1.18056125**	**CNC_021165.1.513801**
3	DFF	4	*qdff-01*	5.06	−0.8135	12.72	0.3016	182.18	191.5	CNC_021163.1.27315241	CNC_021163.1.38343874
		**4**	***qdff-02***	**5.27**	−**0.6712**	**10.39**	**0.2731**	**532.51**	**545.63**	**CNC_021163.1.11351447**	**CNC_021163.1.12812015**
		**6**	***qdff-03***	**3.22**	**0.6502**	**8.20**	**0.2972**	**321.15**	**328.78**	**CNC_021165.1.18056125**	**CNC_021165.1.513801**
		6	*qdff-04*	3.08	0.6634	7.28	0.2870	364.35	374.26	CNC_021165.1.36994104	CNC_021165.1.17940395
4	DHF	4	*qdhf-01*	4.71	−0.7461	10.07	0.2689	182.18	191.5	CNC_021163.1.27315241	CNC_021163.1.38343874
		**4**	***qdhf-02***	**3.40**	−**0.5411**	**6.66**	**0.2602**	**532.51**	**545.63**	**CNC_021163.1.11351447**	**CNC_021163.1.12812015**
5	NPP	4	*qnpp-01*	3.06	2.0760	10.31	0.3384	205.75	218.92	CNC_021163.1.33772884	CNC_021163.1.30731371
		4	*qnpp-02*	3.40	−1.8962	8.58	0.2594	398	406.09	CNC_021163.1.29479703	CNC_021163.1.25311228
		6	*qnpp-03*	3.38	2.1303	9.66	0.2666	2.00	18.75	CNC_021165.1.1002514	CNC_021165.1.8008006
		7	*qnpp-04*	3.94	2.1311	8.05	0.2368	188.69	195.37	CNC_021166.1.23023466	CNC_021166.1.17171266
6	BIO	6	*qbio-01*	3.75	2.5243	7.73	0.2633	2.00	18.75	CNC_021165.1.1002514	CNC_021165.1.8008006
		6	*qbio-02*	3.77	−2.2020	7.42	0.2592	276.16	284.06	CNC_021165.1.21676871	CNC_021165.1.32146805
		7	*qbio-03*	4.02	2.6998	7.92	0.2558	188.69	195.37	CNC_021166.1.23023466	CNC_021166.1.17171266
7	YLD	4	*qyld-01*	4.41	1.9979	13.71	0.3145	205.75	218.92	CNC_021163.1.33772884	CNC_021163.1.30731371
		4	*qyld-02*	4.33	1.7325	10.19	0.2794	218.92	223.15	CNC_021163.1.30731371	CNC_021163.1.30731330
		4	*qyld-03*	3.56	2.0964	13.81	0.3167	532.51	545.63	CNC_021163.1.11351447	CNC_021163.1.12812015
		4	*qyld-04*	3.55	1.5264	7.00	0.2486	547.09	548.02	CNC_021163.1.12812016	CNC_021163.1.12811959
		7	*qyld-05*	3.42	1.6487	6.86	0.3160	188.69	195.37	CNC_021166.1.23023466	CNC_021166.1.17171266
8	HSW	6	*qhsw-01*	3.35	−0.4827	6.72	0.2324	276.16	284.06	CNC_021165.1.21676871	CNC_021165.1.32146805
		7	*qhsw-02*	4.94	0.8518	15.29	0.3074	177.97	188.69	CNC_021166.1.12612605	CNC_021166.1.23023466
9	HI	4	*qhi-01*	4.16	0.9921	11.42	0.2919	205.75	218.92	CNC_021163.1.33772884	CNC_021163.1.30731371
		**4**	***qhi-02***	**4.65**	**0.9668**	**10.55**	**0.2832**	**218.92**	**223.15**	**CNC_021163.1.30731371**	**CNC_021163.1.30731330**
		4	*qhi-03*	3.27	0.7656	6.41	0.2646	547.09	548.02	CNC_021163.1.12812016	CNC_021163.1.12811959
10	MPI	4	*qmpi-01*	3.73	−0.9711	8.96	0.2520	218.92	223.15	CNC_021163.1.30731371	CNC_021163.1.30731330
		**6**	***qmpi-02***	**3.89**	−**1.2668**	**14.77**	**0.3027**	**2.00**	**18.75**	**CNC_021165.1.1002514**	**CNC_021165.1.8008006**
11	RLWC	**4**	***qrlwc-01***	**3.78**	**0.3861**	**8.45**	**0.2641**	**218.92**	**223.15**	**CNC_021163.1.30731371**	**CNC_021163.1.30731330**
		6	*qrlwc-02*	3.01	−0.3107	5.89	0.2399	276.16	284.06	CNC_021165.1.21676871	CNC_021165.1.32146805

DG, days to germination; DFI, days to flowering initiation; DFF, days to 50% flowering; DHF, days to 100% flowering; PH, plant height (cm); NPP, number of pods per plant; BIO, biomass per plant (gm); YLD, yield per plant (gm); HSW, 100-seed weight (gm); HI, harvest index (%); MPI, membrane permeability index; RLWC, relative leaf water content (%); Chr, chromosome number; LOD, logarithm of odds; R^2^, proportion of the variance explained by genetic effect; TR^2^, proportion of the total variance explained by the model including covariates. Bold characters show QTLs, which were common at both locations, i.e., Ludhiana as well as Faridkot.

The italic values provided indicates the names assigned to QTLs identified for different traits, in the present study.

**TABLE 6:** Summary of QTLs associated with various morphological and physiological traits evaluated Faridkot.

S. no.	Trait	Chr	QTL name	LOD	Additive effect	R^2^ (%)	TR^2^	Left flanking marker position (cM)	Right flanking marker position (cM)	Left flanking marker	Right flanking marker
1	DG	2	*qdg-01*	3.66	0.0394	9.15	0.2053	287.19	302.05	CNC_021161.1.17610977	CNC_021161.1.36182232
2	DFI	2	*qdfi-01*	4.48	−0.6750	18.64	0.3936	232.67	250.56	CNC_021161.1.9957038	CNC_021161.1.3423481
		4	*qdfi-02*	4.16	−0.5626	11.04	0.2915	159.6	170.46	CNC_021163.1.29661315	CNC_021163.1.29493473
		**4**	***qdfi-03***	**5.15**	−**0.6576**	**17.11**	**0.3459**	**532.51**	**545.63**	**CNC_021163.1.11351447**	**CNC_021163.1.12812015**
		**6**	***qdfi-04***	**4.12**	**0.5661**	**11.02**	**0.3002**	**321.15**	**328.78**	**CNC_021165.1.18056125**	**CNC_021165.1.513801**
3	DFF	2	*qdff-01*	4.16	−0.7216	17.44	0.3850	232.67	250.56	CNC_021161.1.9957038	CNC_021161.1.3423481
		4	*qdff-02*	4.01	−0.6127	10.71	0.2901	159.6	170.46	CNC_021163.1.29661315	CNC_021163.1.29493473
		**4**	***qdff-03***	**5.56**	−**0.7497**	**18.19**	**0.3520**	**532.51**	**545.63**	**CNC_021163.1.11351447**	**CNC_021163.1.12812015**
		**6**	***qdff-04***	**3.97**	**0.6107**	**10.45**	**0.2957**	**321.15**	**328.78**	**CNC_021165.1.18056125**	**CNC_021165.1.513801**
4	DHF	2	*qdhf-01*	4.00	−0.7213	16.94	0.3838	232.67	250.56	CNC_021161.1.9957038	CNC_021161.1.3423481
		4	*qdhf-02*	3.86	−0.6051	10.11	0.2867	159.6	170.46	CNC_021163.1.29661315	CNC_021163.1.29493473
		**4**	***qdhf-03***	**5.65**	−**0.7584**	**18.18**	**0.3456**	**532.51**	**545.63**	**CNC_021163.1.11351447**	**CNC_021163.1.12812015**
		6	*qdhf-04*	3.98	0.6220	10.49	0.2964	321.15	328.78	CNC_021165.1.18056125	CNC_021165.1.513801
5	NPP	2	*qnpp-01*	3.64	−1.8546	10.36	0.2316	162.41	178.09	CNC_021161.1.24009817	CNC_021161.1.30341279
6	YLD	6	*qyld-01*	3.11	−1.2866	6.52	0.1959	276.16	284.06	CNC_021165.1.21676871	CNC_021165.1.32146805
7	HSW	2	*qhsw-01*	3.69	−0.3816	9.18	0.2351	162.41	178.09	CNC_021161.1.24009817	CNC_021161.1.30341279
8	HI	**4**	***qhi-01***	**3.52**	**0.8841**	**8.26**	**0.2520**	**218.92**	**223.15**	**CNC_021163.1.30731371**	**CNC_021163.1.30731330**
		6	*qhi-02*	3.44	−0.7563	6.83	0.2344	276.16	284.06	CNC_021165.1.21676871	CNC_021165.1.32146805
		6	*qhi-03*	3.01	−0.9501	7.66	0.2551	364.35	374.26	CNC_021165.1.36994104	CNC_021165.1.17940395
9	MPI	**6**	***qmpi-01***	**4.44**	−**1.3652**	**21.05**	**0.3508**	**2.00**	**18.75**	**CNC_021165.1.1002514**	**CNC_021165.1.8008006**
		6	*qmpi-02*	3.89	0.8194	8.12	0.2137	276.16	284.06	CNC_021165.1.21676871	CNC_021165.1.32146805
		7	*qmpi-03*	3.22	−1.0739	10.17	0.2589	177.97	188.69	CNC_021166.1.12612605	CNC_021166.1.23023466
10	RLWC	**4**	***qrlwc-01***	**4.16**	**1.7789**	**13.61**	**0.2518**	**218.92**	**223.15**	**CNC_021163.1.30731371**	**CNC_021163.1.30731330**

DG, days to germination; DFI, days to flowering initiation; DFF, days to 50% flowering; DHF, days to 100% flowering; PH, plant height (cm); NPP, number of pods per plant; BIO, biomass per plant (gm); YLD, yield per plant (gm); HSW, 100-seed weight (gm); HI, harvest index (%); MPI, membrane permeability index; RLWC, relative leaf water content (%); Chr, chromosome number; LOD, logarithm of odds; R^2^, proportion of the variance explained by genetic effect; TR^2^, proportion of the total variance explained by the model including covariates. Bold characters show QTLs, which were common at both locations, i.e., Ludhiana as well as Faridkot.

The italic values provided indicates the names assigned to QTLs identified for different traits, in the present study.

A total of eight consensus QTLs were identified for DFI, DFF, DHF, HI, MPI, and RLWC at both the locations evaluated. Nine QTL clusters containing QTLs for DG, DFI, DFF, DHF, NPP, BIO, YLD, HSW, HI, MPI, and RLWC traits evaluated at Ludhiana were identified on linkage groups CaLG04, CaLG06, and CaLG07. Furthermore, seven QTL clusters were identified containing QTLs for DFI, DFF, DHF, NPP, YLD, HSW, HI, MPI, and RLWC traits evaluated at Faridkot on linkage groups CaLG02, CaLG04, and CaLG06. Four QTL clusters were identified on linkage groups CaLG04 and CaLG06 for traits evaluated at both the locations and contained QTLs for DG, DFI, DFF, DHF, BIO, YLD, HSW, HI, MPI, and RLWC traits. All of these QTLs were distributed on linkage groups CaLG02, CaLG04, CaLG06, and CaLG07, while linkage groups CaLG01, CaLG03, CaLG05, and CaLG08 did not contain any QTL for any location. Maximum QTLs were present on linkage group CaLG04 followed by linkage group CaLG06. The highest phenotypic variation was observed for DG (18.37%) at Ludhiana and MPI (21.05%) at Faridkot. The highest LOD value was observed for DFI (6.85) at Ludhiana and DFF (5.56) at Faridkot. The QTLs having positive or negative additive effects for a particular trait implied that an increase in the proportion of the phenotypic variation of that particular trait was contributed by the allele from GPF 2 or ILWC 292, respectively.

**FIGURE 2:**
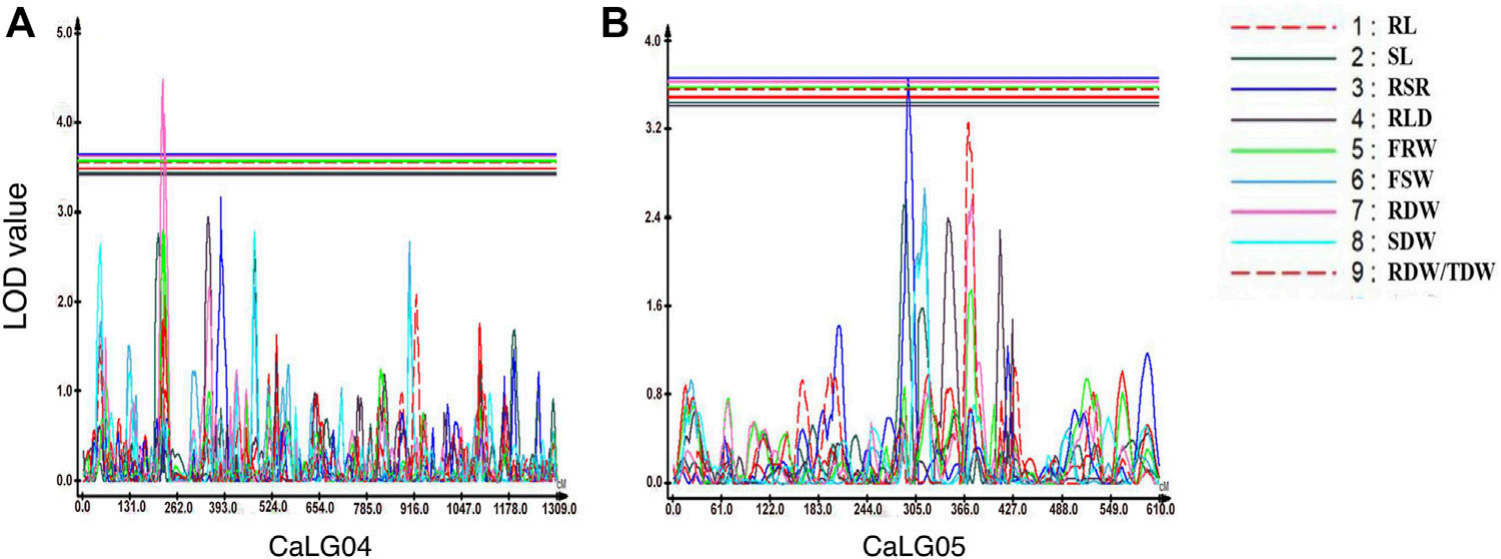
Genomic regions with major-effect QTLs for root-related traits. **(A)** QTLs for root to shoot ratio (RSR) and root dry weight (RDW) were mapped on CaLG04. **(B)** QTLs for root to shoot ratio (RSR) and the ratio of root dry weight to total plant dry weight (RDW/TDW) were mapped on CaLG05.

In the case of root-related traits, a total of seven QTLs were identified for four traits, namely, RSR, RLD, RDW, and RDW/TDW, evaluated under irrigated and rainfed conditions (Table 7; Figure 2; Supplementary Figure S4). Of these, two were major-effect QTLs and five were minor-effect QTLs. One QTL cluster located on linkage group CaLG04 for RDW also contained QTLs for YLD, HI, MPI, and RLWC on the same genomic position. A maximum number of QTLs (four QTLs) for root-related traits were observed for RSR on four different linkage groups. All of these QTLs linked with root-related traits were distributed on linkage groups CaLG02, CaLG04, CaLG05, CaLG06, and CaLG07, while linkage groups CaLG01, CaLG03, and CaLG08 harbored no QTL. The highest phenotypic variation was observed for RDW (11.56%), and the highest LOD value was observed for RLD (5.13).

**TABLE 7:** Summary of QTLs associated with the identified root-related traits under drought stress.

S. no.	Trait	Ch	QTL name	LOD	Additive effect	R^2^ (%)	TR^2^	Left flanking marker position (cM)	Right flanking marker position (cM)	Left flanking marker	Right flanking marker
1	RSR	2	*qrsr-01*	3.54	−0.1946	7.18	0.2598	64.75	74.75	CNC_021161.1.14160111	CNC_021161.1.28928681
		4	*qrsr-02*	3.19	−0.1620	6.17	0.2564	380.66	381.55	CNC_021163.1.32600157	CNC_021163.1.32600103
		5	*qrsr-03*	3.67	0.2083	7.18	0.2568	289.68	294.26	CNC_021164.1.32536050	CNC_021164.1.32971044
		6	*qrsr-04*	3.66	0.2203	9.32	0.2825	463.55	473.88	CNC_021165.1.46709195	CNC_021165.1.52150911
2	RLD	7	*qrld-01*	5.13	0.6382	10.99	0.2170	144.63	145.96	CNC_021166.1.17179431	CNC_021166.1.17179406
3	RDW	4	*qrdw-01*	4.50	0.3234	11.56	0.2545	218.92	223.15	CNC_021163.1.30731371	CNC_021163.1.30731330
4	RDW/TDW	5	*qrdwtdw-01*	3.26	−0.0262	8.89	0.1742	362.47	371.31	CNC_021164.1.3036101	CNC_021164.1.6394203

RSR, root-shoot ratio; RLD, root length density; RDW, root dry weight; RDW/TDW, ratio of root dry weight to total plant dry weight; Ch, chromosome number; LOD, logarithm of odds; R^2^, proportion of the variance explained by genetic effect; TR^2^, proportion of the total variance explained by the model including covariates.

The italic values provided indicates the names assigned to QTLs identified for different traits, in the present study.

#### QTLs for phenological traits

In the case of phenological traits, two QTLs each were detected for DG, DFI, and DHF, while four QTLs were identified for DFF at Ludhiana, having PVE in the range of 6.41–18.37%. Likewise, one QTL for DG and four QTLs each for DFI, DFF, and DHF were identified at Faridkot, having 9.15–18.64% PVE.

#### QTLs for physiological traits

In the case of physiological traits, two QTLs each were identified for MPI and RLWC traits evaluated at Ludhiana, with a PVE of 5.89–14.71%. In addition, three QTLs were detected for MPI and one QTL for RLWC at Faridkot location and had PVE in the range of 8.12–21.05%.

#### QTLs for yield and yield-related traits

Yield and yield-related traits contained two QTLs for HSW, three QTLs each for BIO and HI, four QTLs for NPP, and five QTLs for YLD evaluated at Ludhiana, with a PVE ranging from 6.41% to 15.29%. In addition, one QTL each for NPP, HSW, and YLD and three QTLs for HI were identified for phenotypic data collected at Faridkot, which possessed PVE in the range of 6.52–10.36%.

#### QTLs for root-related traits

In the case of root-related traits, one QTL each for RLD, RDW, and RDW/TDW and four QTLs for RSR were identified, which had PVE ranging from 6.17% to 11.56%.

## Discussion

Drought represents one of the most significant abiotic stresses that causes up to a 60% reduction in chickpea yields (Kumar et al., 2015; Hajjarpoor et al., 2018; Barmukh et al., 2022). Drought tolerance is highly influenced by several parameters such as days to germination, days to 50% flowering, days to maturity, biomass, and yield as morphological traits; transpiration efficiency, membrane permeability, and relative leaf water content as physiological traits; and root depth, root length density, root weight, and root to shoot ratio as root-related traits (Varshney et al., 2011). Due to these factors and unknown mechanisms underlying drought tolerance, molecular mapping for drought tolerance is complicated. Therefore, for genetic dissection of drought tolerance, evaluation of drought tolerance component traits and identification of molecular markers tightly linked to these traits will facilitate the selection of drought-tolerant genotypes and eventually introgression of these traits into elite cultivars through genomics-assisted breeding programs. Thus, it is imperative to study the complex nature of drought stress for identifying key genomic regions associated with drought tolerance component traits for the selection of drought-tolerant genotypes.

In this study, drought stress significantly affected all of the measured morphological, physiological, and root-related traits, except HI, RL, RLD, and FRW. A total of 17 lines were found to be promising for yield and yield-related traits as well as root-related traits under rainfed conditions. Out of these 17 lines, five lines showing early flowering and better yield contributing traits are being evaluated under multi-location trials in India. It has been found in earlier studies that traits such as early flowering, phenological plasticity, and a profuse and deep root system could be beneficial under drought stress (Saxena, 2003; Berger et al., 2006), which is in accordance with our results. Early flowering can be a good option as more pods are set before the occurrence of drought stress, and thus genotypes can escape drought stress (Kushwah et al., 2020b). Breeding programs for developing drought-tolerant genotypes in chickpea were focused on accelerating flowering and maturity to escape terminal drought stress (Upadhyaya et al., 2012). The primary adaptive strategy identified by several chickpea breeding programs for tolerance to terminal drought stress is drought escape *via* early flowering (Kumar & Abbo, 2001; Berger et al., 2006; Gaur et al., 2008; Purushothaman et al., 2014) in spite of the fact that selection pressure for drought tolerance and drought escape *per se* are different from each other (Berger et al., 2016). However, some previous studies have observed that early flowering is negatively correlated with seed yield under drought stress (Kumar et al., 2005; Yucel et al., 2006). Hence, there is a need to develop drought-tolerant, high-yielding chickpea genotypes with early maturity.

Previous studies show that root traits, such as root length density, root depth, and root dry weight, could be promising for improving drought tolerance in chickpea (Kashiwagi et al., 2006; Zaman-Allah et al., 2011; Purushothaman et al., 2017; Barmukh et al., 2022). The present study also showed that root length density was non-significantly affected by drought stress. Prolific root systems are likely to influence transpiration, biomass, and harvest index under drought conditions through the utilization of deep soil moisture (Zaman-Allah et al., 2011; Kashiwagi et al., 2013; Purushothaman et al., 2016). Despite the significance of prolific root systems for drought stress, only a few advances have been made in this direction, mainly because root studies are laborious and time consuming. Shoot and root vigor are reciprocally advantageous as the production of shoot biomass depends on the exploitation of soil moisture by the root system (Pinheiro et al., 2005; Purushothaman et al., 2017; Sivasakthi et al., 2018) and root vigor depends on the production of photo-assimilates by shoots (Wasson et al., 2014). This suggests that further improvements in root-related traits could advance drought stress tolerance in chickpeas as higher yields and harvest index can be attained with a strong root system. A high correlation coefficient was observed between the irrigated and rainfed conditions for all root-related traits except RLD. Our results are in accordance with a previous study (Zaman-Allah et al., 2011) in which RLD did not differ between sensitive and tolerant chickpea genotypes under drought stress and had no correlation with seed yield.

In the present study, the pooled ANOVA for all measured traits, including root-related traits, showed highly significant differences between genotypes under irrigated and rainfed conditions at both locations. Combined ANOVA in several studies also showed significant differences for various morphological and physiological traits (Hamwieh et al., 2013; Pang et al., 2017; Purushothaman et al., 2017; Sachdeva et al., 2018) and for root-related traits (Varshney et al., 2014; Purushothaman et al., 2017). Significant differences were also observed for genotype × environment (G × E) interactions for almost all the measured traits including root-related traits. Both the locations have almost similar trends of rainfall patterns under rainfed conditions with little differences. Thus, significant G × E interaction could be due to other factors other than the available soil moisture. To encounter these differences, BLUP values for genotypes for both the locations were also estimated taking location as a random effect. BLUP values of the RIL population for both locations showed high correlations with each, thus showing that these can be used for further QTL analysis to find consistent QTLs at both the locations. BLUPs were also estimated from phenotypic data across the years and locations by several researchers for statistical analysis and QTL mapping (Thudi et al., 2014; Li et al., 2018; Sivasakthi et al., 2018).

Due to polygenic control and high G × E interaction, quantitative traits like drought tolerance are complex in nature. Because of this, little progress could be made to breed cultivars harboring these traits through conventional breeding approaches. Thus, identifying the QTLs for complex drought tolerance component traits is an important requirement for understanding their genetic architecture and precise transfer in the background of elite cultivars. In the present study, QTLs having a positive additive effect indicated that the donor parent allele (GPF 2) contributed toward increasing the trait value; while those with a negative additive effect indicated that the recipient parent allele (ILWC 292) conferred a higher trait value. A total of 31 QTLs at Ludhiana and 23 QTLs at Faridkot were identified for the 12 morphological and physiological traits excluding root-related traits using BLUPs in the RIL population evaluated under irrigated and rainfed conditions. Out of these, eight consensus QTLs for DFI, DFF, DHF, HI, MPI, and RLWC were identified at both locations. QTLs responsible for early flowering have an advantage of more pod setting before the occurrence of drought stress due to a comparatively longer period of reproductive growth. Early flowering genotypes follow the mechanism of drought escape by reducing the duration of crop maturity under drought stress (Krishnamurthy et al., 2010; Purushothaman et al., 2016), which indicated that this trait is a useful selection criterion for drought tolerance. The consensus QTLs for DFI, DFF, and DHF harbor on linkage groups 4 and 6, suggesting that these loci confer flowering time in chickpea.

A total of three QTLs and two QTLs for seed yield were reported earlier by Jingade and Ravikumar (2015) and Rehman et al. (2011), respectively, on CaLG01. Likewise, one QTL on CaLG04 (Cobos et al., 2007) and four QTLs on linkage groups CaLG04, CaLG06, CaLG07, and CaLG08 (Verma et al., 2015) for seed yield were also identified. Some QTLs for seed yield were also mapped in the present study, which were located on linkage groups CaLG04, CaLG06, and CaLG07. Similarly, two QTLs on CaLG04 and CaLG08 (Cobos et al., 2009), two QTLs on CaLG06 and CaLG07 (Gupta et al., 2015), and one QTL on CaLG04 (Jamalabadi et al., 2013) for seed weight were identified. Recently, major-effect QTLs for seed weight were identified on CaLG06 and CaLG04 in chickpea (Barmukh et al., 2021b). In the present study, three QTLs were identified for seed weight on linkage groups CaLG02, CaLG06, and CaLG07. Several QTLs for plant height, number of pods per plant, 100-seed weight, biomass, harvest index, and yield were also identified in some previous studies (Gowda et al., 2011; Varshney et al., 2014) which were also at the same locus as identified in our study.

Solute leakage from the cell membrane is used to estimate the damage caused by drought stress (ElBasyoni et al., 2017). Drought-tolerant genotypes show less cell membrane damage, and thus, the membrane permeability index can be used as an effective selection criterion against drought stress. Likewise, RLWC indicates the equilibrium between water content in the leaf tissues and transpiration rate (Lugojan & Ciulca, 2011). Thus, RLWC is also a key indicator of water status present in plants and helps in the efficient selection of drought-tolerant genotypes. Consequently, QTLs representing genotypic differences in the RIL population for both MPI and RLWC traits can be used in genomics-assisted breeding programs for improving drought tolerance in chickpea.

While roots represent the first plant parts to be exposed to drought conditions in the soil, improvement of root-related traits holds the potential to enhance soil water extraction under drought scenarios. Breeding strategies should concentrate on the improvement of root-related traits for the efficient utilization of available soil water. In the present study, a total of seven QTLs were identified for four root-related traits, namely, RSR, RLD, RDW, and RDW/TDW, in the RIL population evaluated under irrigated and rainfed conditions. In the past, Varshney and colleagues (2014) identified robust main-effect QTLs for root traits such as root length density, root surface area, and root dry weight/total plant dry weight ratio, explaining up to 16.67% PVE. Furthermore, Thudi et al. (2014) identified 15 significant molecular markers closely associated with root length density, root dry weight, rooting depth, root surface area, and root volume. Due to the lack of precise and high-throughput phenotyping for root-related traits, breeding for the advancement of drought tolerance appears to be a difficult task. In the present study, molecular mapping of root-related traits will be helpful for the introgression of the genomic regions associated with root traits into elite cultivars by using genomics-assisted breeding approaches (Varshney et al., 2021).

Four QTL clusters were consistently identified at both Ludhiana and Faridkot locations, containing QTLs for DG, DFI, DFF, DHF, BIO, YLD, HSW, HI, MPI, and RLWC at the same genomic position on CaLG04 and CaLG06. One QTL cluster located on CaLG04 possessed QTLs for RDW, YLD, HI, MPI, and RLWC traits. Several genomic regions having co-localized/pleiotropic QTLs can be scrutinized to identify closely linked molecular markers, which will be helpful for introgressing this region into elite cultivars through marker-assisted breeding programs. Previously, Varshney et al. (2014) identified a total of nine QTL clusters for drought tolerance-related traits, out of which one major cluster on CaLG04 was referred to as a “*QTL hotspot*”. Importantly, the *“QTL-hotspot”* region was found to contain 13 major QTLs for 12 drought tolerance component traits and explained up to 58.20% PVE (Varshney et al., 2014). The estimated size of this “*QTL hotspot*” was refined from 29 cM to 14 cM using a genotyping-by-sequencing approach (Jaganathan et al., 2015) and then to ∼300 kb using a bin mapping approach (Kale et al., 2015). Notably, fine mapping of the *“QTL-hotspot”* region led to the identification of *CaTIFY4b* as the candidate gene regulating drought responses in chickpea (Barmukh et al., 2022). In the recent past, introgression of the “*QTL-hotspot*” region into different genetic backgrounds of elite cultivars led to the development and release of molecular breeding lines with enhanced drought tolerance (Varshney et al., 2013b; Bharadwaj et al., 2021). In a similar way, the QTL clusters identified in the present study can be targeted for introgression into elite cultivars for enhancing drought stress tolerance. These identified QTLs will serve as a potential tool for detecting candidate genes with the recent advances in genomics and transcriptomics resources in chickpea.

## Data Availability

The raw sequencing reads of RIL population and its parents has been deposited in NCBI-SRA and is accessible publically through the BioProject ID: PRJNA530673 and the submission ID: SUB5404800.

## Supplementary material

Supplementary Figure S1 Scatter plots of drought tolerance-related traits showing a relationship between Ludhiana and Faridkot locations. FDK, Faridkot; LDH, Ludhiana.

Supplementary Figure S2 Scatter plots of root-related traits showing a relationship between Ludhiana and Faridkot locations. FDK, Faridkot; LDH, Ludhiana.

Supplementary Figure S3 Genomic regions with QTLs for morphological and physiological traits. For traits evaluated at Faridkot, (**A**) QTLs for days to germination (DG), days to flowering initiation (DFI), days to 50% flowering (DFF), days to 100% flowering (DHF), number of pods per plant (NPP), and 100-seed weight (HSW) were mapped on CaLG02; while (**B**) QTL for membrane permeability index (MPI) was mapped on CaLG07. (**C**) QTLs for days to germination (DG), number of pods per plant (NPP), biomass per plant (BIO), yield per plant (YLD), and 100-seed weight (HSW) were mapped on CaLG07 for Ludhiana.

Supplementary Figure S4 Genomic regions with QTLs for root-related traits. QTLs for root to shoot ratio (RSR) mapped on (**A**) CaLG02 and (**B**) CaLG06. (**C**) QTL for root length density (RLD) mapped on CaLG07.
