# Supplementary material for: Genetic mapping of QTLs for drought tolerance in chickpea (Cicer arietinum L.)
Source: Front Genet. 2022 Aug 19;13:953898. doi: 10.3389/fgene.2022.953898 (PMC9437436; doi:10.3389/fgene.2022.953898)
Supplement: Supplementary file 1 [file DataSheet1.pdf]

Genetic mapping of QTLs for drought tolerance in chickpea (*Cicer arietinum* L.)

Ashutosh Kushwah[1], Dharminder Bhatia[1], Rutwik Barmukh[2], Inderjit Singh[1], Gurpreet Singh[3], Shayla Bindra[1], Suruchi Vij[1], Bharadwaj Chellapilla[4], Aditya Pratap[5], Manish Roorkiwal[2,#], Shiv Kumar[6], Rajeev K. Varshney[2,7*], Sarvjeet Singh[1*]

[1]Department of Plant Breeding & Genetics, Punjab Agricultural University, Ludhiana, Punjab, India-141004

[2]Center of Excellence in Genomics and Systems Biology, International Crops Research Institute for the Semi-Arid Tropics (ICRISAT), Hyderabad, India

[3]Regional Research Station, Punjab Agricultural University, Faridkot, India

[4]Division of Genetics, ICAR-Indian Agricultural Research Institute, New Delhi, India

[5]Crop Improvement Division, ICAR- Indian Institute of Pulses Research, Kanpur, India

[6]International Center for Agricultural Research in the Dry Areas (ICARDA), Rabat Office, Rabat, Morocco

[7]Murdoch's Centre for Crop & Food Innovation, State Agricultural Biotechnology Centre, Food Futures Institute, Murdoch University, Murdoch, Western Australia, Australia

[#]Present address: Khalifa Center for Genetic Engineering and Biotechnology, United Arab Emirates University, Al-Ain, United Arab Emirates

*Correspondence:

rajeev.varshney@murdoch.edu.au (Rajeev K. Varshney);

sarvjeet62@pau.edu (Sarvjeet Singh)

Supplementary Figures

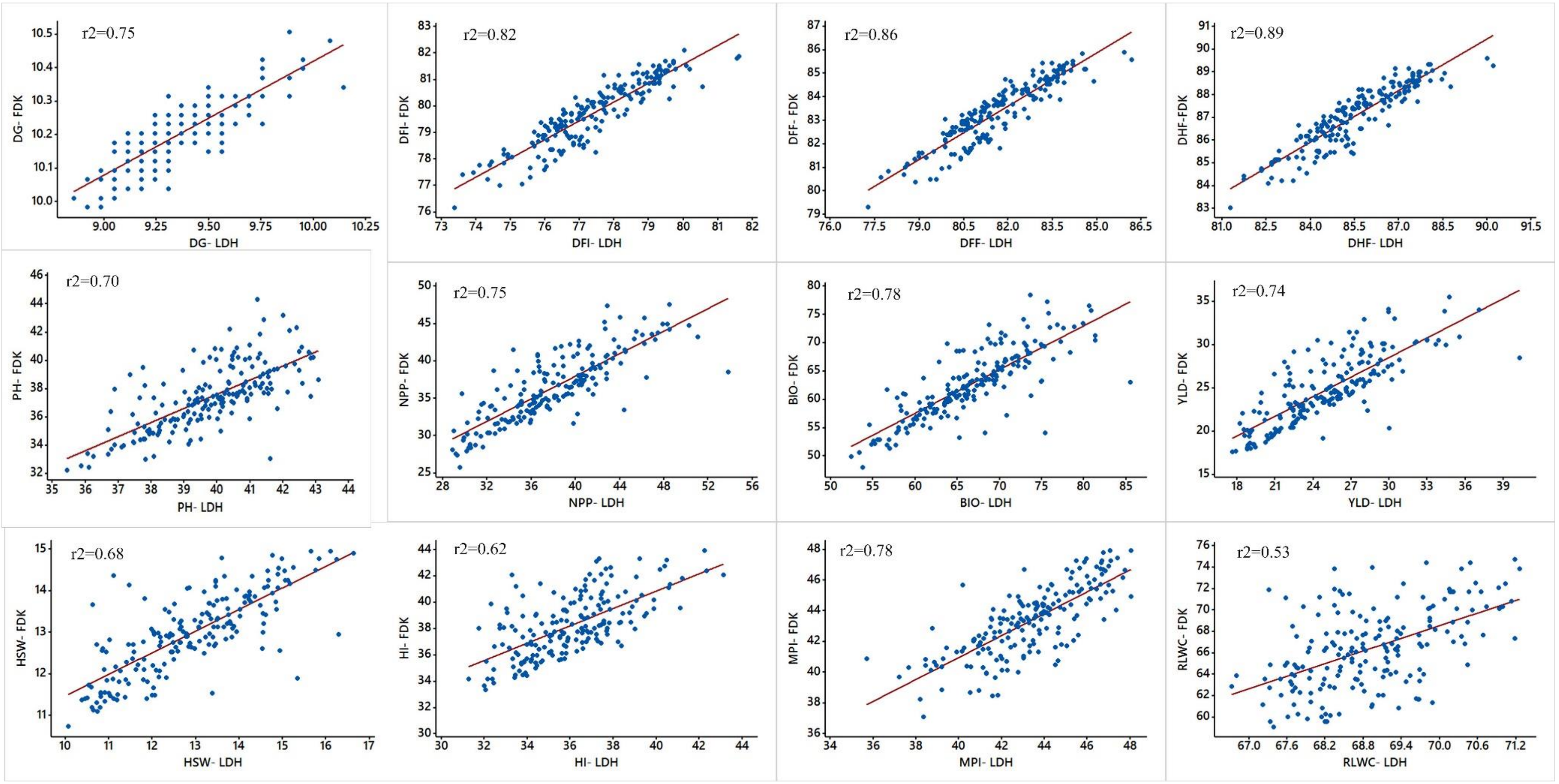

Supplementary Figure S1: The scatter plots of drought tolerance related traits showing relationship between Ludhiana and Faridkot locations. FDK, Faridkot; LDH, Ludhiana.

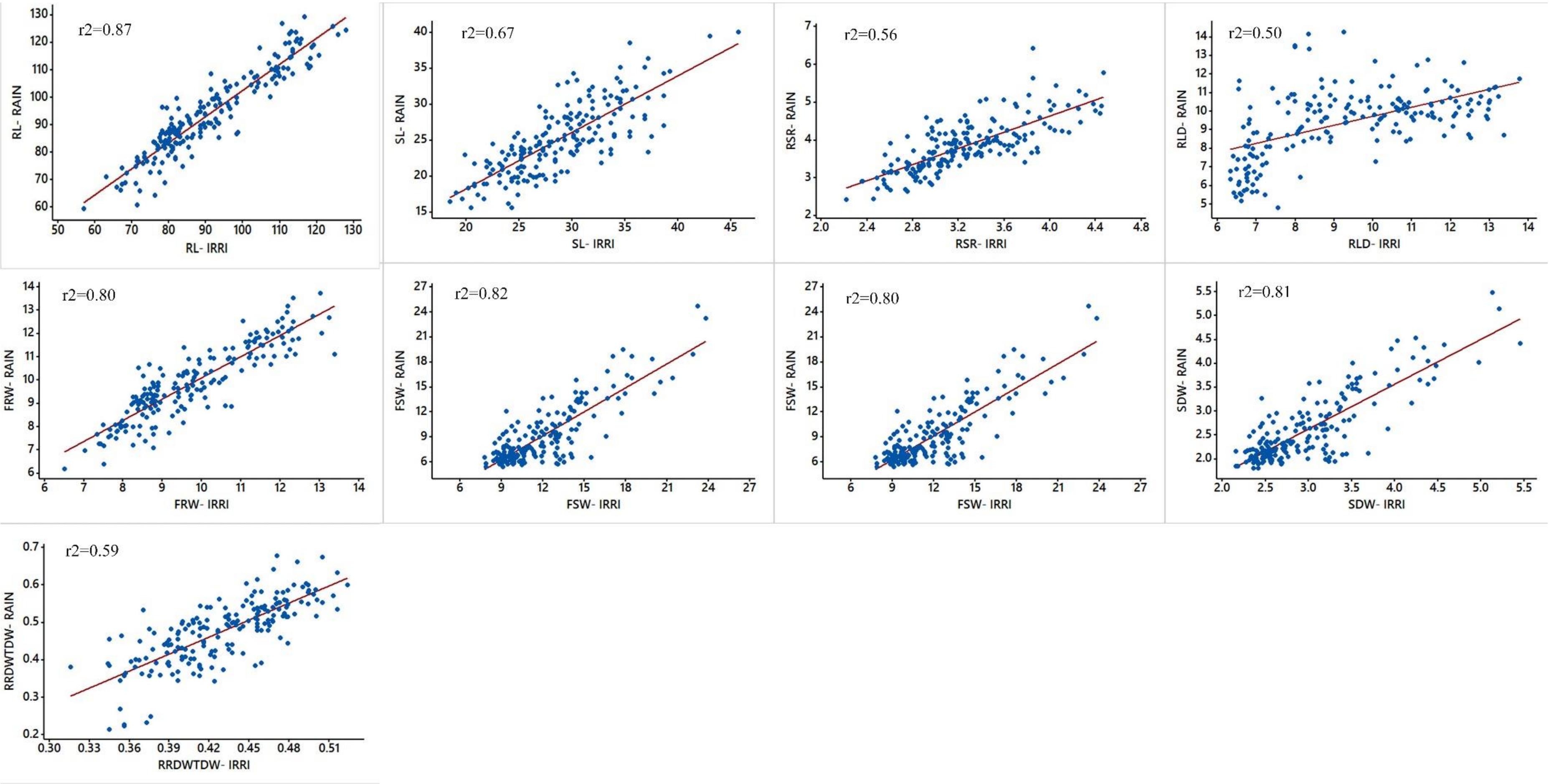

Supplementary Figure S2: The scatter plots of root-related traits showing relationship between Ludhiana and Faridkot locations. FDK, Faridkot; LDH, Ludhiana.

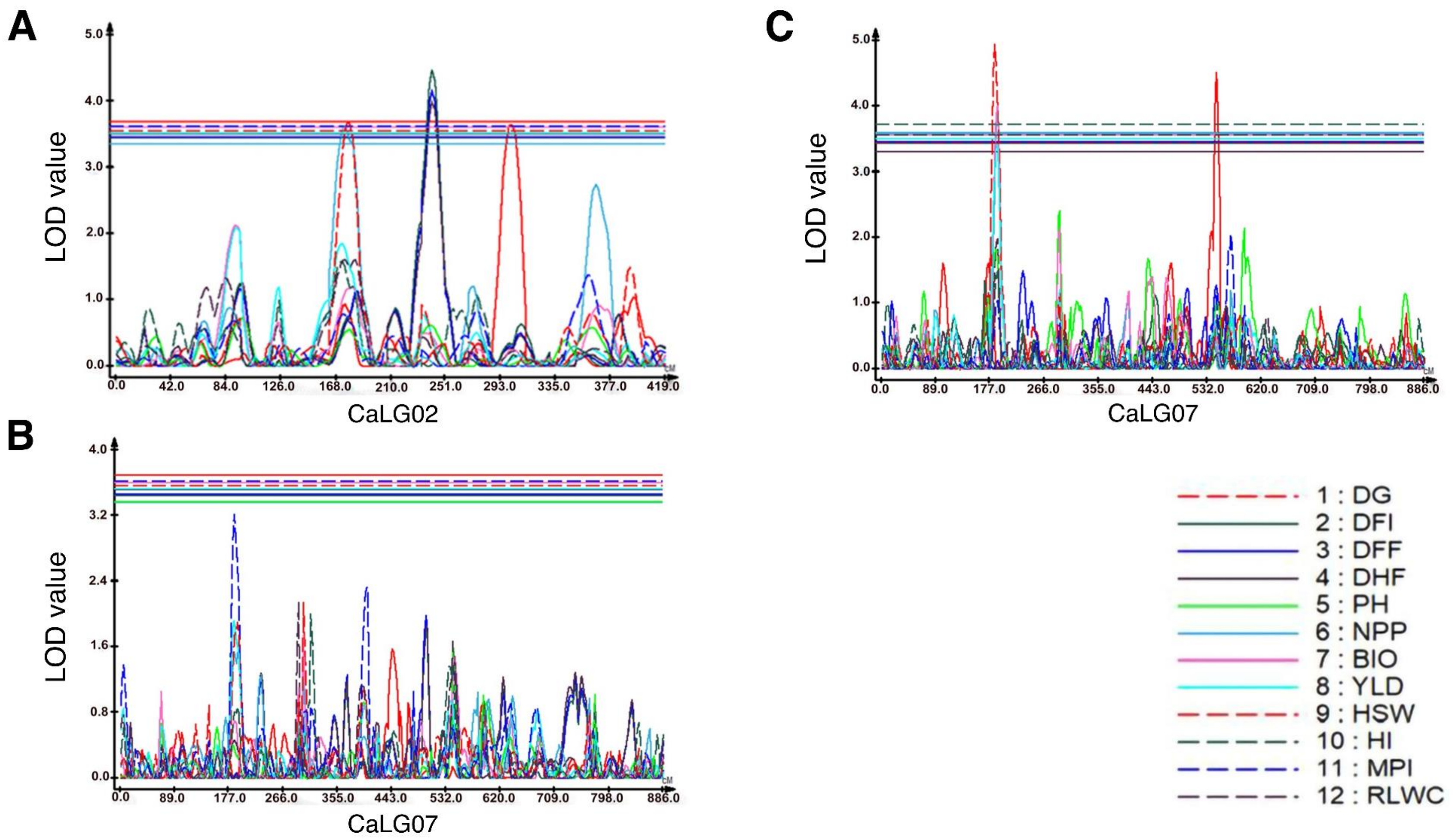

Supplementary Figure S3: Genomic regions with QTLs for morphological and physiological traits. For traits evaluated at Faridkot. (**A**) QTLs for days to germination (DG), days to flowering initiation (DFI), days to 50% flowering (DFF), days to 100% flowering (DHF), Number of pod per plant (NPP), and 100-seed weight (HSW) were mapped on CaLG02; while (**B**) QTL for membrane permeability index (MPI) was mapped on CaLG07. (**C**) QTLs for days to germination (DG), Number of pod per plant (NPP), biomass per plant (BIO), yield per plant (YLD), and 100-seed weight (HSW) were mapped on CaLG07 for Ludhiana.

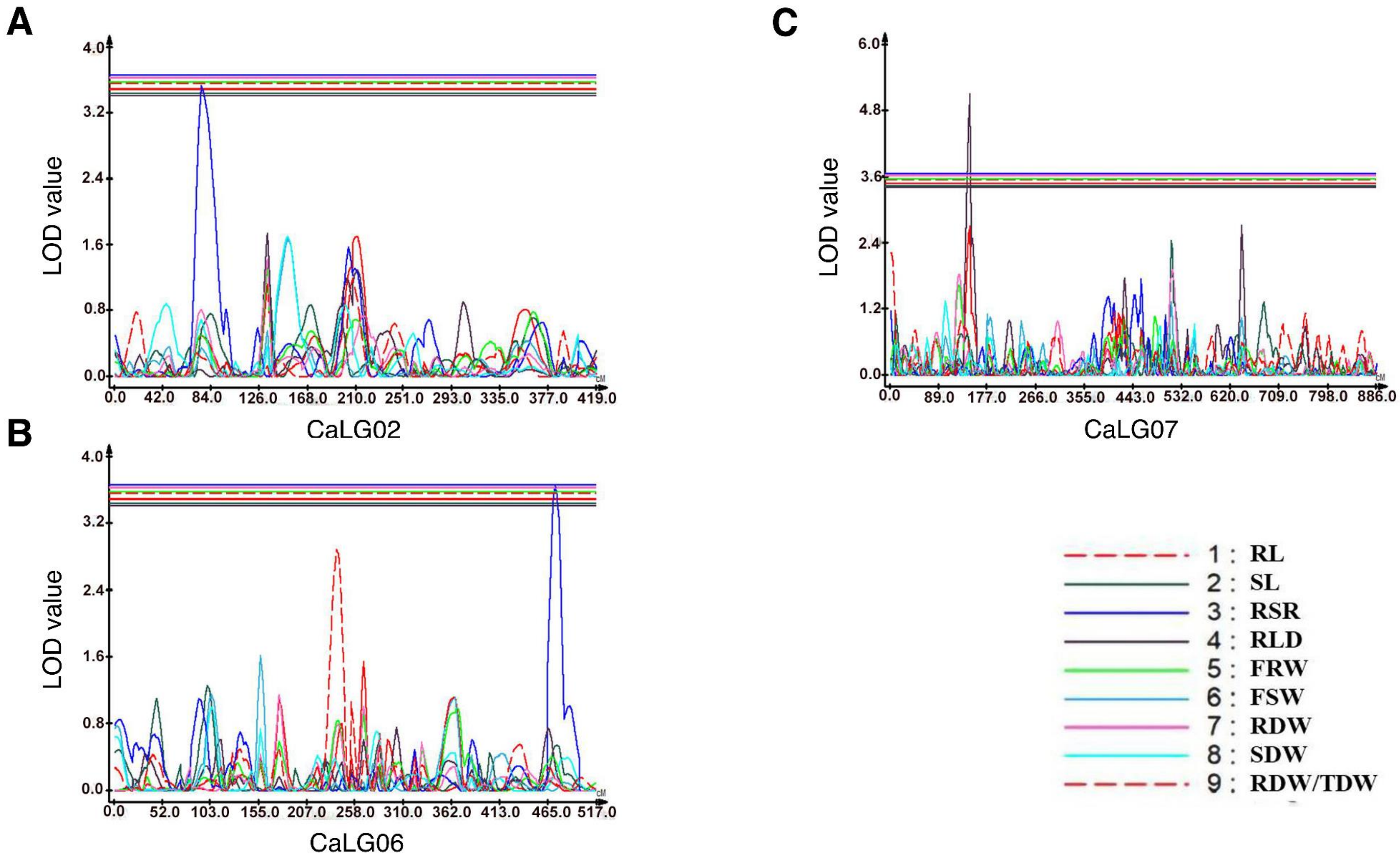

Supplementary Figure S4: Genomic regions with QTLs for root-related traits. QTLs for root-shoot ratio (RSR) mapped on (**A**) CaLG02 and (**B**) CaLG06. (**C**) QTL for root length density (RLD) mapped on CaLG07.
